# Transcranial Magnetic Stimulation to Assess Exercise-Induced Neuroplasticity

**DOI:** 10.3389/fnrgo.2021.679033

**Published:** 2021-05-31

**Authors:** Claudia V. Turco, Aimee J. Nelson

**Affiliations:** Department of Kinesiology, McMaster University, Hamilton, ON, Canada

**Keywords:** TMS, neurophysiology, aerobic exercise, corticospinal excitability, SICI

## Abstract

Aerobic exercise facilitates neuroplasticity and has been linked to improvements in cognitive and motor function. Transcranial magnetic stimulation (TMS) is a non-invasive technique that can be used to quantify changes in neurophysiology induced by exercise. The present review summarizes the single- and paired-pulse TMS paradigms that can be used to probe exercise-induced neuroplasticity, the optimal stimulation parameters and the current understanding of the neurophysiology underlying each paradigm. Further, this review amalgamates previous research exploring the modulation of these paradigms with exercise-induced neuroplasticity in healthy and clinical populations and highlights important considerations for future TMS-exercise research.

## Introduction

Exercise-induced neuroplasticity refers to the change in the nervous system that occurs following an acute or chronic bout of exercise. These neuroplastic effects of exercise are exerted at the molecular, cellular and structural levels of the nervous system, which accumulate to induce changes in brain function (El-Sayes et al., 2019a). Transcranial magnetic stimulation (TMS) is a non-invasive technique that can be used to quantify subtle changes in brain excitation and inhibition that accompany exercise.

**Figure 1:**
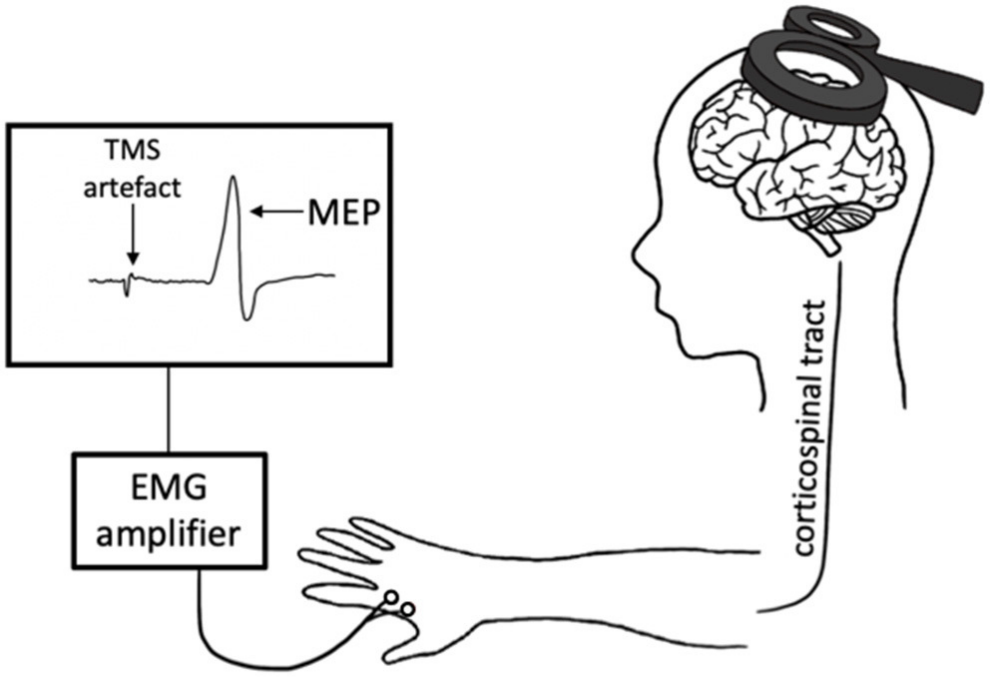
Transcranial magnetic stimulation (TMS) schematic. An electric current is generated in the TMS coil, producing a magnetic field perpendicular to the coil. This magnetic field passes through the scalp, inducing a perpendicular secondary electric field within the cortex. This electric current depolarizes the cortical neurons in M1, evoking a descending volley along the corticospinal tract and subsequently a motor-evoked potential (MEP) in the target muscle. This response is measured with electromyography (EMG).

Transcranial magnetic stimulation produces a magnetic field that depolarizes superficial pyramidal neurons within the cortex. When delivered to the primary motor cortex (M1), TMS trans-synaptically activates corticospinal output neurons, evoking a descending corticospinal volley that results in a motor response within the target muscle (Hallett, 2007). This muscle response, known as the motor-evoked potential (MEP), is then quantified with surface electromyography (EMG) (Figure 1). There are numerous TMS paradigms that can be used to study different aspects of neurophysiology including cortical excitability, motor neuron recruitment and, indirectly, neurotransmitter receptor function. As such, TMS provides a unique opportunity to non-invasively probe the neuronal mechanisms that underpin exercise-induced neuroplasticity.

The goal of this review is to guide users on the application of TMS for evaluating the neurophysiological mechanisms underlying exercise-induced neuroplasticity and to provide a comprehensive review of the literature that have used TMS to evaluate exercise-induced neuroplasticity. Specifically, we will discuss single- and paired-pulse TMS paradigms that are valuable for quantifying neurophysiological changes that accompany aerobic exercise. We will also discuss the use of these techniques in previous research that explored exercise-induced neuroplasticity in healthy and clinical populations. Within the context of this review, acute exercise refers to a single session of exercise while chronic exercise refers to multiple sessions of exercise or aerobic training.

## Single-Pulse TMS Paradigms

Epidural recordings have provided insight into the neural populations activated by a single pulse of TMS when delivered to M1. TMS depolarizes superficial cortical layers, leading to trans-synaptic activation of the pyramidal output neurons and evoking a series of descending volleys known as indirect waves (I-waves) that are labeled in order of appearance (I1–I4) (Di Lazzaro et al., 2012). Only at very high intensities is TMS able to directly activate deeper pyramidal output neurons in M1, evoking a direct wave (D-wave) in the epidural space (Di Lazzaro et al., 2012). Modulation of I-waves provides valuable insight into the physiology underlying TMS paradigms, as discussed below. Table 1 shows how single-pulse TMS paradigms reportedly change following aerobic exercise.

**Table 1:** Variation in TMS measures with aerobic exercise.

**Study**	**Exercise**	**RMT**	**AMT**	**MEP**	**CSP**	**iSP**	**SICI**	**ICF**	**LICI**	**SICF**	**SAI**	**LAI**	**CBI**	**IHI**
Andrews et al. (2020)	MICE (50% HRR) HIIE (90% HRR)	– –	–	ϕ ϕ	–	– –	ϕ ϕ	ϕ ϕ	ϕ ϕ	– –	– –	– –	– –	– –
Brown et al. (2020)	MICE (65–70% HR_max_)	–	–	ϕ	–	–	–	–	–	–	ϕ	↑	–	–
El-Sayes et al. (2020)	MIIE (60–79% HR_max_) HIIE (80–100% HR_max_)	ϕ ϕ	–	ϕ ϕ	– –	– –	– –	– –	– –	– –	– –	– –	– –	– –
Moscatelli et al. (2020)^*^	MIAT (60–75% HR_max_)	↓	–	↓	–	–	–	–	–	–	–	–	–	–
Nicolini et al. (2020)	HIIE (105–125% W_peak_)	ϕ	ϕ	↑	–	–	ϕ	ϕ	–	–	–	–	–	–
El-Sayes et al. (2019b)	MICE (65–70% HR_max_)	ϕ	ϕ	↑	–	–	↓	–	–	–	–	–	–	–
MacDonald et al. (2019)	LICE (30% HRR) MICE (40–50% HRR)	– –	– –	ϕ ↑	–	– –	– –	– –	– –	– –	– –	– –	– –	– –
Morris et al. (2020)	LICE (40–60% HRR)	ϕ	–	ϕ	–	–	ϕ	↑	ϕ	–	–	–	–	–
Nicolini et al. (2019)^*^	HIIT (105–135% W_peak_)	–	–	ϕ	–	–	ϕ	↓	–	–	–	–	–	–
Opie and Semmler (2019)	LICE (50% HRR) HIIE (77% HRR)	– –	–	↑ ↑	–	– –	ϕ ↓	– –	–	– –	–	– –	–	– –
Walsh et al. (2019)	MICE (RPE 10–12)	–	–	ϕ	–	–	–	–	–	–	–	–	–	–
Yamazaki et al. (2019)	LICE (30% VO_2peak_)	ϕ	–	ϕ	–	–	↓	ϕ	ϕ	ϕ	↓	–	–	–
McGregor et al. (2018)^*^	MICE (50–75% HRR)	ϕ	–	–	–	↑	–	–	–	–	–	–	–	ϕ
Smith et al. (2018)	HICE (80% HRR)	–	–	ϕ	–	–	–	–	–	–	–	–	–	–
Lulic et al. (2017)	MICE (50–70% HR_max_)	ϕ	ϕ	↑/ϕ^#^	–	–	↓	↓	–	ϕ	–	–	–	–
Neva et al. (2017)	MICE (65–70% HR_max_)	–	–	ϕ	↓	↓	–	–	–	↑	–	–	–	–
Stavrinos and Coxon (2017)	HIIE (90% HRR)	–	–	ϕ	–	–	↓	–	ϕ	–	–	–	–	–
Singh et al. (2016)	MICE (60–75% HR_max_)	–	–	ϕ	–	–	–	–	–	–	–	–	–	–
Mang et al. (2016)	HIIE (90% W_peak_)	–	–	–	–	–	–	–	–	–	–	–	↓	–
Mooney et al. (2016)	MICE (73% HRR)	–	ϕ	ϕ	ϕ	–	ϕ	–	↓	–	–	–	–	–
Mang et al. (2014)	HIIE (90% W_peak_)	–	–	ϕ	–	–	–	–	–	–	–	–	–	–
Singh et al. (2014)	MICE (65–70% HR_max_)	–	–	ϕ	–	–	↓	↑	ϕ	–	–	–	–	–
Smith et al. (2014)	LICE (40% HRR) HICE (80% HRR)	ϕ ϕ	– –	ϕ ϕ	– –	– –	↓ ↓	– –	– –	– –	– –	– –	– –	– –
McDonnell et al. (2013)	LICE (55–65% HR_max_) MICE (75% HR_max_)	– –	– –	ϕ ϕ	– –	– –	– –	– –	– –	– –	– –	– –	– –	– –

* *Indicates chronic aerobic exercise (i.e., long-term training). All other studies represent an acute session of aerobic exercise*.

# *Indicates MEPs increased in the high fit group only and did not change in the low fit group. ϕ indicates no change following exercise, ↑ indicates an increase following exercise, and ↓ indicates a decrease following exercise*.

*AMT, active motor threshold; CBI, cerebellar inhibition; CSP, cortical silent period; HICE, high intensity continuous exercise; HIIE, high intensity interval exercise; HIIT, high intensity interval training; HR_max_, maximal heart rate; HRR, heart rate reserve; ICF, intracortical facilitation; IHI, interhemispheric inhibition; iSP, ipsilateral silent period; LAI, long-latency afferent inhibition; LICE, low intensity continuous exercise; LICI, long-interval intracortical inhibition; MEP, motor-evoked potential; MIAT, moderate intensity aerobic training; MICE, moderate intensity continuous exercise; MIIE, moderate intensity interval exercise; RMT, resting motor threshold; RPE, rating of perceived exertion; SAI, short-latency afferent inhibition; SICF, short-interval intracortical facilitation; SICI, short-interval intracortical inhibition; VO_2peak_, peak oxygen uptake; W_peak_, peak power output*.

### Motor Threshold

Resting motor threshold (RMT) refers to the lowest intensity of TMS that evokes a MEP. RMT represents excitation of the lowest threshold neurons within M1 and is therefore an assessment of baseline cortical excitability. It is increased by drugs that block voltage-gated Na^+^ channels (Ziemann et al., 1996b; Chen et al., 1997; Boroojerdi et al., 2001; Sommer et al., 2012; Lang et al., 2013), suggesting that RMT reflects axonal excitability. For example, a low RMT suggests a lower threshold for activation of neurons within M1, or greater cortical excitability.

Several approaches exist to quantify RMT. There is the traditional Rossini-Rothwell method proposed by Rossini et al. (1994) and later revised (Rothwell et al., 1999) that uses a systematic approach to estimate RMT. This approach may require up to ~50 trials to accurately determine RMT (Mills and Nithi, 1997; Mishory et al., 2004). Alternatively, more automated approaches can be used to estimate RMT with similar accuracy and reliability, but with fewer trials required. For example, the adaptive threshold-hunting method based on maximum likelihood parameter estimation by sequential testing (ML-PEST) predicts the TMS intensity that yields a 50% probability of evoking an MEP of at least 50 μV (Awiszus, 2003). This approach requires only 20 trials to reliably estimate RMT (Ah Sen et al., 2017). In addition, a Bayesian adaptive method has been shown to require as little as 7 trials to estimate RMT (Qi et al., 2011).

Active motor threshold (AMT) refers to the lowest intensity of TMS that evokes a MEP of at least 200 μV with a probability of 50%, while the target muscle is isometrically contracted at 10–20% of the maximum voluntary contraction (MVC) (Groppa et al., 2012). The AMT is typically lower than RMT since descending commands for voluntary contraction partially activate the upper and lower motor neurons of the corticospinal tract. Therefore, less TMS intensity is needed to bring the corticospinal tract to threshold and evoke a MEP. Similar to RMT, AMT can be estimated with systematic or automated approaches, where automated approaches require fewer trials for accurate estimation (Ah Sen et al., 2017).

Previous research has shown that an acute bout of aerobic exercise does not change RMT (Smith et al., 2014; Lulic et al., 2017; Yamazaki et al., 2019; El-Sayes et al., 2020; Morris et al., 2020; Nicolini et al., 2020) or AMT (Lulic et al., 2017; Nicolini et al., 2020). Similarly, in chronic stroke, a single session of either high-intensity interval exercise (HIIE) or moderate intensity continuous exercise (MICE) does not alter RMT (Abraha et al., 2018), while HIIE reduces AMT but MICE does not (Boyne et al., 2019). Eight weeks of aerobic training reduced RMT in healthy controls (Moscatelli et al., 2020), while 12-weeks of aerobic cycling did not change RMT in older adults (McGregor et al., 2018) and 4-weeks of treadmill training increased RMT in Parkinson's disease (PD) (Yang et al., 2013). This may suggest that acute aerobic exercise is not potent enough to influence the lowest threshold neurons within M1, while chronic aerobic exercise is. Although the literature suggests that RMT and AMT are typically unchanged following acute aerobic exercise, these results may reflect the specific and age-range of the participants tested. We therefore advise research to measure RMT and AMT in the event that changes in cortical excitability following exercise occur within the specific population tested. Further, acquisition of these metrics will still be required to adjust stimulation intensities for single- and paired-pulse TMS paradigms as described below.

### Motor-Evoked Potentials

**Figure 2:**
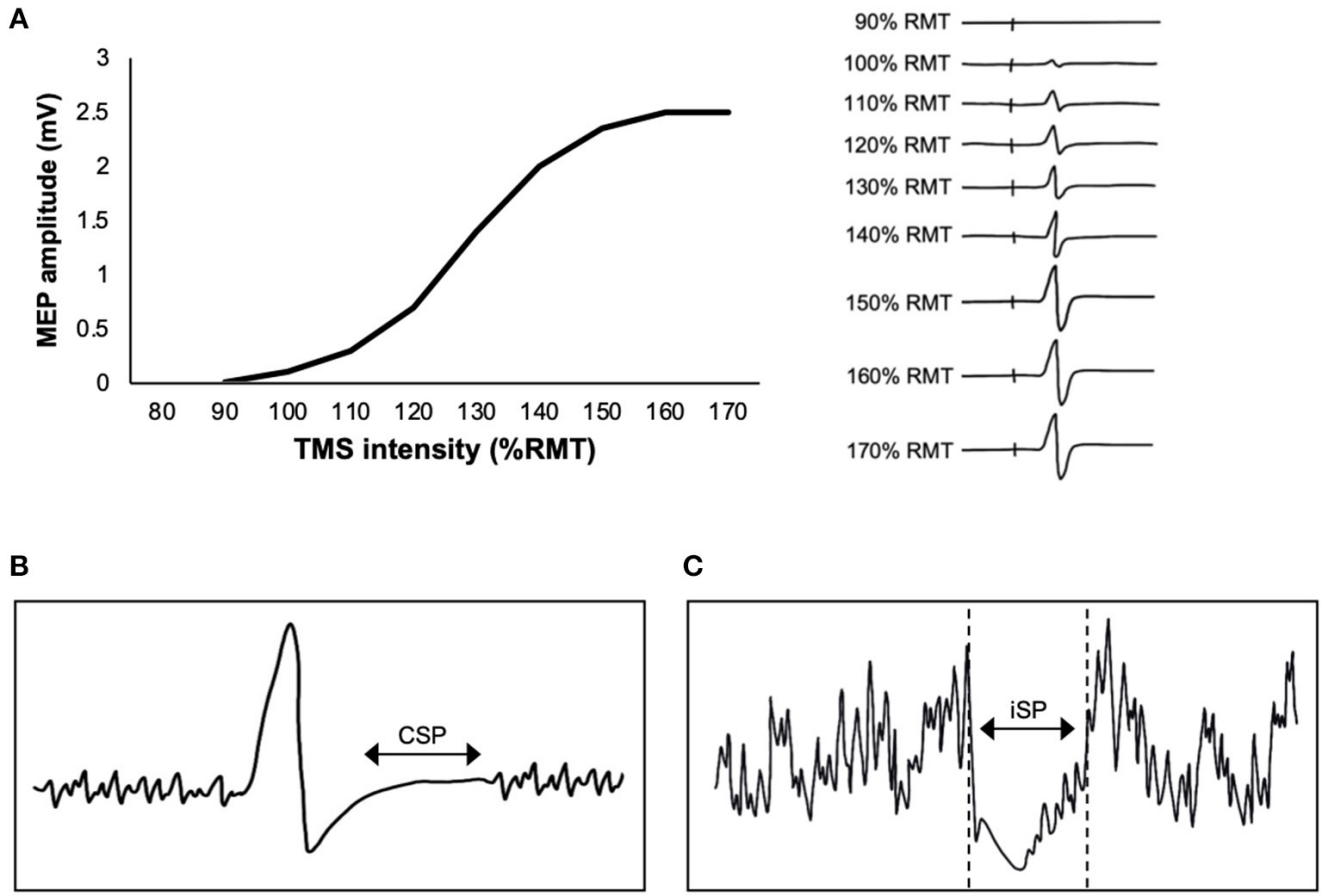
Single-pulse transcranial magnetic stimulation (TMS) paradigms. **(A)** Graph (left) shows an example of a motor-evoked potential (MEP) recruitment curve, plotting the relationship between TMS intensity and MEP amplitude. The individual MEPs (right) increase in amplitude as the TMS intensity is increased, reflecting recruitment of motor neurons within the corticospinal tract. **(B)** Schematic of the contralateral silent period (CSP), where EMG activity is suppressed immediately following a TMS pulse delivered to M1 during contraction of the target muscle (a muscle contralateral to TMS). **(C)** Schematic of the ipsilateral silent period (iSP), where EMG activity is suppressed immediately following a TMS pulse delivered to M1 during contraction of a muscle that is ipsilateral to TMS.

It is common to report the peak-to-peak MEP amplitude derived from a single TMS intensity. However, MEP recruitment curves capture the changing relationship between TMS intensity and MEP amplitude, providing a more comprehensive understanding of the effects of exercise. As TMS intensity is increased, MEP amplitude increases in a sigmoidal fashion (Devanne et al., 1997) (Figure 2A). The x-intercept of the curve reflects RMT, the rising slope reflects the rate of upper and lower motor neuron recruitment, the plateau at higher TMS intensities reflects recruitment of most motor neurons within the corticospinal tract, and the area under the MEP recruitment curve reflects corticospinal excitability. The slope of the MEP recruitment curve is also positively correlated with glutamate levels in M1 as assessed via magnetic resonance spectroscopy (MRS) (Stagg et al., 2011). Therefore, MEP recruitment curves provide unique information about motor neuron recruitment and glutamatergic neurotransmission compared to a single MEP. To construct a MEP recruitment curve, MEPs are obtained as a function of RMT or maximum stimulator output (MSO) (Boroojerdi et al., 2001), where the average peak-to-peak amplitude of minimum 5 MEPs (Cavaleri et al., 2017) is obtained in increments of 10% RMT or 10% MSO. For example, previous TMS exercise studies have acquired MEP recruitment curves using intensities ranging from 90 to 200% RMT in increments of 10% RMT (Mang et al., 2014; Singh et al., 2014; Smith et al., 2014; Lulic et al., 2017; Neva et al., 2017; Stavrinos and Coxon, 2017; El-Sayes et al., 2019b, 2020; MacDonald et al., 2019; Walsh et al., 2019; Brown et al., 2020; Nicolini et al., 2020). Studies have also obtained MEP recruitment curves during active contraction of 10% MVC and using intensities ranging from 90 to 200% AMT (Lulic et al., 2017; Nicolini et al., 2019, 2020).

The effects of acute aerobic exercise on corticospinal excitability in healthy individuals are mixed. Studies have reported either an increase (Lulic et al., 2017; El-Sayes et al., 2019b; MacDonald et al., 2019; Opie and Semmler, 2019; Nicolini et al., 2020) or no change (McDonnell et al., 2013; Mang et al., 2014, 2016; Singh et al., 2014, 2016; Smith et al., 2014, 2018; Mooney et al., 2016; Neva et al., 2017; Stavrinos and Coxon, 2017; Walsh et al., 2019; Yamazaki et al., 2019; Andrews et al., 2020; Brown et al., 2020; El-Sayes et al., 2020; Morris et al., 2020) in corticospinal excitability following a single session of exercise. In these studies, corticospinal excitability was assessed with either single-pulse MEPs (McDonnell et al., 2013; Mang et al., 2016; Mooney et al., 2016; Singh et al., 2016; Smith et al., 2018; Opie and Semmler, 2019; Yamazaki et al., 2019; Andrews et al., 2020; Morris et al., 2020) or MEP recruitment curves (Mang et al., 2014; Singh et al., 2014; Smith et al., 2014; Lulic et al., 2017; Neva et al., 2017; Stavrinos and Coxon, 2017; El-Sayes et al., 2019b, 2020; MacDonald et al., 2019; Walsh et al., 2019; Brown et al., 2020; Nicolini et al., 2020). Further, studies have shown that an acute bout of aerobic exercise increases the area under the MEP recruitment curve when obtained during rest only but not active conditions (Lulic et al., 2017; Nicolini et al., 2020).

These studies assessing the effects of acute aerobic exercise used a range of exercise intensities (high, moderate, low), exercise patterns (interval, continuous) and cardiorespiratory fitness or physical activity inclusion criteria (sedentary, moderately active, highly active). However, the relationship between corticospinal excitability and these factors are unclear. One study showed that aerobic fitness was not related to the magnitude of corticospinal excitability change following exercise (MacDonald et al., 2019). Further, it was suggested that only highly-active participants showed an increase in corticospinal excitability following acute aerobic exercise (El-Sayes et al., 2020), however, our recent study showed an increase in corticospinal excitability within sedentary participants (Nicolini et al., 2020). Alternatively, an interaction between fitness and exercise intensity may exist such that higher intensity exercise is required to induce neuroplasticity in sedentary individuals. For example, several studies show no change in MEPs within low-fit individuals with moderate (Lulic et al., 2017; El-Sayes et al., 2020) and high (Stavrinos and Coxon, 2017; El-Sayes et al., 2020) intensity exercise. However, Nicolini et al. (2020) found an increase in MEPs within sedentary males when exercised at a high workload of 105–125% peak power output (W_peak_). This is in contrast to El-Sayes et al. (2020) where a lower workload of ~69% W_peak_ was used in the HIIE protocol.

In stroke, one session of low-intensity cycling did not change MEPs within the first dorsal interosseous (FDI) muscle (Murdoch et al., 2016). However, one session of HIIE does increase MEPs in the extensor carpi radialis (ECR) muscle when TMS is delivered to the lesioned hemisphere, but not the non-lesioned hemisphere (Li et al., 2019). Further, one session of HIIE also increases MEPs in the non-paretic tibialis anterior (TA) muscle, but decreases MEPs in the paretic TA (Madhavan et al., 2016). Overall, these results show that high-intensity but not low-intensity exercise is sufficient to modulate corticospinal excitability in stroke. However, the direction of corticospinal excitability changes following a single session of HIIE is mixed. Therefore, further research will be needed to determine whether HIIE is capable of increasing corticospinal output to paretic musculature. Further, these results were acquired immediately following the exercise session, and it is unknown how long changes in corticospinal excitability persist.

The effects of chronic aerobic exercise on corticospinal excitability in healthy individuals is also mixed. Moscatelli et al. (2020) showed that MEP amplitude increases in untrained males following 12 weeks of moderate intensity aerobic training, and Nicolini et al. (2019) showed no change in MEP recruitment curves following 6 weeks of high-intensity interval training (HIIT) in sedentary males. At present, it is unknown whether females would exhibit a change in corticospinal excitability following long-term aerobic training, or if moderate-highly active individuals would respond to long-term aerobic training. In incomplete spinal cord injury (SCI), 3–5 months of aerobic treadmill training increased corticospinal excitability (Thomas and Gorassini, 2005). Specifically, there was an increase in the plateau of the MEP recruitment curve, MEP_max_, and MEPs at the half-way point of the MEP recruitment curve. This suggests that chronic aerobic exercise induces short-term plasticity that may contribute to restoring the motor output in SCI.

### Contralateral Silent Period (CSP)

Transcranial magnetic stimulation delivered to M1 during voluntary activation produces a period of EMG suppression directly following the MEP (Figure 2B). The length of this period is referred to as the contralateral silent period (CSP) (Cantello et al., 1992). The CSP is typically 100–300 ms in length (Säisänen et al., 2008), and reflects a combination of both intracortical and spinal mechanisms (Škarabot et al., 2019). The H-reflex is suppressed within the first 50–80 ms of the CSP (Ziemann et al., 1993). Further, the CSP is only >100 ms in length when evoked by TMS delivered to M1 but ~50 ms when evoked by electrical stimulation of the cervicomedullary junction (Inghilleri et al., 1993). These findings suggest that the earlier portion of the CSP (<80 ms) is mediated by spinal mechanisms, while the later portion (>80 ms) is cortically mediated.

The CSP is observed when the participant maintains isometric contraction of the contralateral target muscle at intensities ranging from 10% (Goodall et al., 2018) to 100% (Mira et al., 2017) of the MVC. However, CSP tends to shorten with greater contraction intensities (Matsugi, 2019). Further, the CSP tends to lengthen with higher TMS intensities until a plateau occurs at very high intensities (~>75% MSO) (Kimiskidis et al., 2005). This suggests that the inhibitory drive of voluntary activation saturates at high enough stimulation intensities. Previous TMS exercise studies have evoked CSP with a TMS intensity of 130% RMT and contraction for 20% MVC (Neva et al., 2017), or with a TMS intensity adjusted to evoked a silent period of 175 ms during 10% MVC (Mooney et al., 2016). Other research has also used the TMS intensity that evokes a 1 mV MEP at rest (Tremblay et al., 2011; Locke et al., 2020).

The CSP is lengthened by gamma-aminobutryic acid (GABA) reuptake inhibitors (Werhahn et al., 1999; Pierantozzi et al., 2004a). It has been hypothesized that CSP is modulated by GABA_B_ receptor activity (Ziemann et al., 2015). Although multiple studies have reported no effect of GABA_B_ receptor agonists on CSP length (Inghilleri et al., 1996; Ziemann et al., 1996b; McDonnell et al., 2006), those that have shown an increase in CSP delivered baclofen intrathecally, bypassing the blood brain barrier that baclofen does not readily cross (Siebner et al., 1998; Stetkarova and Kofler, 2013). Alternatively, oral administration of zolpidem, a positive allosteric modulator of the GABA_A_ receptor, lengthens the CSP (Mohammadi et al., 2006). Therefore, the data suggests that CSP may indirectly reflect both GABA_A_ and GABA_B_ receptor activity.

The CSP is either reduced (Neva et al., 2017) or unchanged (Mooney et al., 2016) following acute aerobic exercise in healthy individuals. In stroke, one session of HIIE and MICE does not change CSP length (Boyne et al., 2019). The effects of chronic aerobic exercise on CSP in healthy individuals has yet to be investigated. However, 4–8 weeks of treadmill training lengthens CSP in PD (Fisher et al., 2008; Yang et al., 2013) and 3–5 months of treadmill training lengthens CSP in SCI (Thomas and Gorassini, 2005). This suggests that long-term aerobic training is needed to consistently induce a change in GABAergic neurotransmission within M1.

### Ipsilateral Silent Period (iSP)

The ipsilateral silent period (iSP) is an assessment of transcallosal inhibition, whereby a suprathreshold pulse of TMS delivered to M1 induces interruption of EMG activity in an ipsilateral muscle (Figure 2C). For example, Neva et al. (2017) delivered TMS at 150% RMT and observed interruption of EMG activity in the ipsilateral abductor pollicis brevis (APB) that was isometrically contracted at 50% MVC. The duration of the iSP is ~30 ms (Perez and Cohen, 2009; Neva et al., 2017). The iSP is thought to be cortically-mediated, as it does not lead to reduction in the H-reflex (Wassermann et al., 1991). To our knowledge, only one study has investigated the effects of acute aerobic exercise on iSP. Neva et al. (2017) found that an acute bout of MICE reduced iSP bilaterally. Only one study has assessed the effects of chronic aerobic exercise on iSP. McGregor et al. (2018) showed that 12 weeks of aerobic cycling increased the duration of the iSP in older adults. Therefore, both acute and chronic aerobic exercise may modulate the excitability of transcallosal fibers.

## Paired-Pulse TMS Paradigms

Transcranial magnetic stimulation can be used to study inhibitory and excitatory mechanisms within the cortex. These techniques involve paired-pulse paradigms, whereby a conditioning stimulus (CS) delivered prior to a test stimulus (TS) modulates the resulting MEP. Parameters including the stimulation intensities and interstimulus intervals (ISIs) can be adjusted to determine the direction of MEP modulation. Modulation of the MEP is then expressed as a ratio of the MEP produced by the CS-TS pair to that produced by the TS alone. A ratio above one denotes facilitation of the MEP, while a ratio below one denotes inhibition of the MEP. Table 1 shows paired-pulse TMS paradigms are reported to change following aerobic exercise.

### Short-Interval Intracortical Inhibition (SICI)

**Figure 3:**
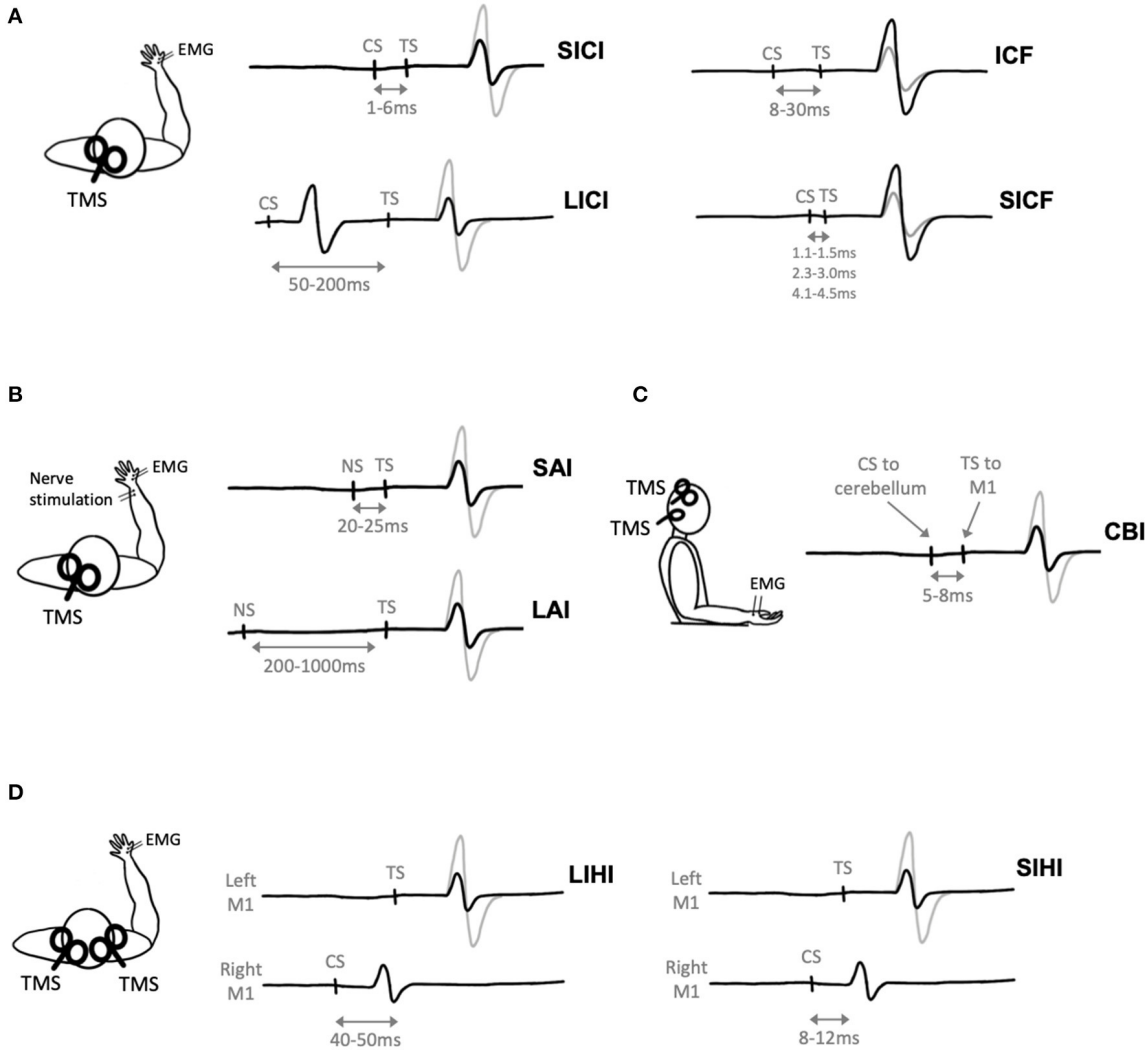
Paired-pulse transcranial magnetic stimulation (TMS) paradigms. Gray electromyography (EMG) traces represent trials where a single TMS pulse is delivered (i.e., test stimulus, TS), black traces represent trials where a conditioning stimulus (CS) and TS are delivered. **(A)** Two TMS pulses delivered in quick succession to M1 evokes short-interval intracortical inhibition (SICI), long-interval intracortical inhibition (LICI), intracortical facilitation (ICF), or short-interval intracortical facilitation (SICF) depending on the interstimulus interval (ISI) between the CS and TS. **(B)** Nerve stimulation (NS) delivered prior to TMS evoked short-latency afferent inhibition (SAI) or long-latency afferent inhibition (LAI) depending on the ISI between the NS and TS. **(C)** Cerebellar inhibition (CBI) is observed when the CS is delivered to the cerebellum 5ms prior to the TS delivered to M1. **(D)** A TMS pulse delivered to the right hemisphere (CS) prior to a pulse delivered to the left hemisphere (TS) evokes short-interval interhemispheric inhibition (SIHI) or long-interval interhemispheric inhibition (LIHI) depending on the ISI between the CS and TS.

Short-interval intracortical inhibition (SICI) is one such paired-pulse paradigm that describes inhibition of the MEP (Figure 3A). Short-interval intracortical inhibition is observed when two TMS pulses are delivered in quick succession, with an ISI between 1 and 6 ms (Kujirai et al., 1993). The ISI of 1 ms may reflect the axonal refractory period (Chan et al., 2002; Fisher et al., 2002) while SICI at ~2–3 ms reflects GABAergic neurotransmission (Di Lazzaro et al., 2005a). It is thought to be cortically mediated, as epidural recordings show that SICI reduces late I3 waves (Di Lazzaro et al., 1998) and does not modulate MEPs evoked by transcranial electric stimulation (TES), which directly activates pyramidal output neurons (Kujirai et al., 1993).

To measure SICI, the first pulse (i.e., CS) is delivered at a sub-threshold intensity while the second pulse (i.e., TS) is delivered at a supra-threshold intensity. Previous TMS exercise studies have evoked SICI using CS intensities of 80–90% AMT (Smith et al., 2014; Mooney et al., 2016; Lulic et al., 2017; Stavrinos and Coxon, 2017; El-Sayes et al., 2019b; Nicolini et al., 2019, 2020; Yamazaki et al., 2019) or 70–90% RMT (Singh et al., 2014; Opie and Semmler, 2019; Morris et al., 2020) and TS intensities of 120% RMT (Singh et al., 2014; Morris et al., 2020) or adjusted to evoked a 1 mV MEP (Smith et al., 2014; Mooney et al., 2016; Lulic et al., 2017; Stavrinos and Coxon, 2017; El-Sayes et al., 2019b; Nicolini et al., 2019, 2020; Opie and Semmler, 2019; Yamazaki et al., 2019; Andrews et al., 2020).

Short-interval intracortical inhibition is mainly thought to indirectly reflect activity of the GABA_A_ receptor. It is upregulated by benzodiazepines, positive allosteric modulators of the GABA_A_ receptor (Di Lazzaro et al., 2005a, 2007), and may specifically involve GABA_A_ receptors containing the α2 or α3-subunits (Di Lazzaro et al., 2007; Teo et al., 2009). Short-interval intracortical inhibition is also reduced by the GABA_B_ agonist baclofen (McDonnell et al., 2006), although other studies show no effect (Ziemann et al., 1996b; McDonnell et al., 2007). Further, SICI is modulated by dopamine (DA) as DA agonists increase SICI (Ziemann et al., 1996a, 1997; Korchounov et al., 2007) and DA antagonists decrease SICI (Ziemann et al., 1997), and by noradrenaline (NA) as NA agonists decrease SICI (Ilić et al., 2003; Gilbert et al., 2006). Overall, the pharmacology of SICI suggests that this measure may reflect the activity of inhibitory interneurons expressing GABA_A_ receptors, under the control of pre-synaptic GABA_B_-receptor mediated inhibition and modulated by DA and NA.

Modulation of SICI with exercise may indicate changes in GABA_A_ receptor activity or the excitability of intracortical interneurons underlying SICI. In healthy controls, acute aerobic exercise either decreases (Singh et al., 2014; Smith et al., 2014; Lulic et al., 2017; Stavrinos and Coxon, 2017; El-Sayes et al., 2019b; Opie and Semmler, 2019; Yamazaki et al., 2019) or does not change SICI (Mooney et al., 2016; Opie and Semmler, 2019; Andrews et al., 2020; Morris et al., 2020; Nicolini et al., 2020). Notably, changes in SICI have only been shown when acquired with ISIs of 2–3 ms (Singh et al., 2014; Smith et al., 2014; Lulic et al., 2017; El-Sayes et al., 2019b; Opie and Semmler, 2019; Yamazaki et al., 2019) whereas SICI acquired with an ISI of 1 ms does not change with exercise (Mooney et al., 2016; Stavrinos and Coxon, 2017). This suggests that exercise may modulate GABA_A_ receptor neurotransmission rather than axonal refractory periods.

In regards to chronic exercise, 6 weeks of HIIT does not change SICI in sedentary healthy males (Nicolini et al., 2019). Further, a single session of aerobic exercise is not sufficient to change SICI in the upper limb of individuals with stroke (Murdoch et al., 2016; Abraha et al., 2018; Li et al., 2019). However, 4 weeks of treadmill training increased lower-limb SICI in PD (Yang et al., 2013). This may suggest that prolonged aerobic exercise is required to modulate intracortical inhibition within clinical populations.

### Intracortical Facilitation (ICF)

A subthreshold CS followed by a suprathreshold TS with an ISI ranging from 8 to 30 ms evokes intracortical facilitation (ICF) (Kujirai et al., 1993) (Figure 3A). Previous TMS exercise studies have evoked ICF using CS intensities of 80–90% AMT (Smith et al., 2014; Mooney et al., 2016; Lulic et al., 2017; Stavrinos and Coxon, 2017; El-Sayes et al., 2019b; Nicolini et al., 2019, 2020; Yamazaki et al., 2019) or 70–90% RMT (Singh et al., 2014; Opie and Semmler, 2019; Morris et al., 2020) and TS intensities of 120% RMT (Singh et al., 2014; Morris et al., 2020) or adjusted to evoked a 1 mV MEP (Smith et al., 2014; Mooney et al., 2016; Lulic et al., 2017; Stavrinos and Coxon, 2017; El-Sayes et al., 2019b; Nicolini et al., 2019, 2020; Opie and Semmler, 2019; Yamazaki et al., 2019; Andrews et al., 2020). The physiological mechanisms of ICF are less well understood, as ICF does not modulate epidural recordings of I-waves (Di Lazzaro et al., 2006). ICF is reduced by N-methyl-D-aspartate (NMDA) receptor antagonists (Ziemann et al., 1998a) and benzodiazepines, positive allosteric modulators of the GABA_A_ receptor (Inghilleri et al., 1996; Ziemann et al., 1996c), but is increased by NA agonists (Moll et al., 2003; Gilbert et al., 2006).

Changes in ICF with exercise may indirectly reflect a change in glutamatergic neurotransmission with M1. In healthy individuals, multiple studies have shown no change in ICF following an acute bout of aerobic exercise (Yamazaki et al., 2019; Andrews et al., 2020; Nicolini et al., 2020) while others show ICF increased (Singh et al., 2014; Morris et al., 2020) and another revealed a decrease (Lulic et al., 2017). Further, a single session of HIIE or MICE does not change ICF in chronic stroke (Abraha et al., 2018). However, 6 weeks of HIIT reduces ICF in sedentary males (Nicolini et al., 2019). Interestingly, only ICF at the ISI of 10 ms has been shown to decrease with exercise (Lulic et al., 2017; Nicolini et al., 2019), while only ICF at the ISI of 12 ms increases with exercise (Singh et al., 2014; Morris et al., 2020). Therefore, we recommend that future studies acquire SICI and ICF at a range of ISIs in an input-output curve fashion to assess the time course of MEP modulation (Kujirai et al., 1993), as the neural mechanisms underlying these measures may differ at distinct ISIs.

### Long-Interval Intracortical Inhibition (LICI)

Long-interval intracortical inhibition (LICI) is observed following a suprathreshold CS and suprathreshold TS with an ISI of 50–200 ms (Valls-Solé et al., 1992) (Figure 3A). Previous TMS exercise studies have evoked LICI using CS and TS intensities of 120% RMT (Singh et al., 2014; Yamazaki et al., 2019; Morris et al., 2020), adjusted to evoke a 1 mV MEP (Stavrinos and Coxon, 2017; Yamazaki et al., 2019; Andrews et al., 2020), or adjusted to evoke a CSP of 175 ms (Mooney et al., 2016). Long-interval intracortical inhibition reduces late I-waves, suggesting that this phenomenon is of cortical origin (Nakamura et al., 1997; Chen et al., 1999; Di Lazzaro et al., 2002). Further, LICI is increased by the GABA_B_ receptor baclofen (McDonnell et al., 2006) and GABA reuptake inhibitors tiagabine (Werhahn et al., 1999) and vigabatrin (Pierantozzi et al., 2004b). However, benzodiazepines do not modulate LICI (Teo et al., 2009). Therefore, LICI may indirectly reflect activation of GABA_B_ receptors.

A change in LICI with exercise-induced neuroplasticity may indicate that exercise modulates activity of the GABA_B_ receptor or neural mechanisms underlying LICI. In healthy individuals, only one study has shown a decrease in LICI following aerobic exercise (Mooney et al., 2016), while majority of studies show no change in LICI (Singh et al., 2014; Smith et al., 2014; Stavrinos and Coxon, 2017; Yamazaki et al., 2019; Andrews et al., 2020; Morris et al., 2020). Alternatively, 4 weeks of treadmill training increases LICI in PD (Yang et al., 2013). Mooney et al. (2016) acquired LICI at longer ISIs of 125, 175, and 200 ms, whereas others used shorter ISIs of 100–120 ms (Yang et al., 2013; Singh et al., 2014; Smith et al., 2014; Stavrinos and Coxon, 2017; Yamazaki et al., 2019; Andrews et al., 2020; Morris et al., 2020). Therefore, these results may suggest that the neural mechanisms underlying LICI at shorter (<120 ms) and longer (>120 ms) ISIs are distinct.

### Short-Interval Intracortical Facilitation (SICF)

Short-interval intracortical facilitation (SICF) occurs following a suprathreshold CS paired with a perithreshold or subthreshold TS at discrete ISIs of 1.1–1.5, 2.3–3.0, and 4.1–4.5 ms (Ziemann et al., 1998b) (Figure 3A). Previous TMS exercise studies have evoked SICF with a CS intensity of 90% RMT and TS intensity adjusted to evoke a 1 mV MEP (Lulic et al., 2017; Neva et al., 2017; Yamazaki et al., 2019).

Short-interval intracortical facilitation facilitates I-waves recorded from the epidural space, suggesting it is exerted intracortically (Di Lazzaro et al., 1999b). It is reduced by drugs that enhance inhibitory neurotransmission including lorazepam, vigabatrin and phenobarbital (Ziemann et al., 1998c). However, SICF may not reflect GABA_B_ receptor activity as it is not modulated by baclofen (Ziemann et al., 1998c). Further, SICF is not modulated by drugs enhancing excitatory neurotransmission including sodium channel blockers and NMDA receptor antagonists (Ziemann et al., 1998c).

Modulation of SICF with exercise would indicate a change in the excitability of I-wave generating interneurons. An acute bout of aerobic exercise has been shown to either increase (Neva et al., 2017) or not change (Lulic et al., 2017; Yamazaki et al., 2019) SICF. At present, it is unknown whether long-term training modulates SICF, or if SICF is modulated following acute or chronic aerobic exercise in clinical populations.

### Afferent Inhibition

Afferent inhibition refers to the suppression of MEPs when a conditioning electrical stimulus is applied to a peripheral nerve prior to TMS delivered to M1 (Figure 3B). Short-latency afferent inhibition (SAI) is observed when peripheral stimulation precedes TMS by 20–25 ms, while long-latency afferent inhibition (LAI) is observed at interstimulus intervals of 200–1,000 ms. Both SAI and LAI can be observed within muscles of the hand following stimulation of cutaneous digital nerves or the mixed ulnar or median nerves at the wrist. Previous TMS exercise studies have evoked SAI and LAI using TS intensities of 1 mV MEP (Yamazaki et al., 2019; Brown et al., 2020) or 120% RMT (Yamazaki et al., 2019), and nerve stimulation intensities corresponding to 3x sensory threshold (Yamazaki et al., 2019) or motor threshold (Brown et al., 2020).

It is well-known that SAI reflects cholinergic activity as it is reduced by the muscarinic antagonist scopolamine (Di Lazzaro et al., 2000) and increased by acetylcholinesterase inhibitors (Di Lazzaro et al., 2005c). In addition, SAI is reduced by lorazepam and zolpidem but not diazepam, suggesting that SAI reflects activity of GABA_A_ receptors containing the α1-subunit (Di Lazzaro et al., 2005b, 2007; Turco et al., 2018). However, SAI is not modulated by the GABA_B_ receptor agonist baclofen (Turco et al., 2018). Finally, NA reuptake inhibitor reboxetine reduces SAI (Kuo et al., 2017), potentially through the inhibition of acetylcholine release (Vizi and Pasztor, 1981). This pharmacological evidence may suggest that SAI is reflective of GABAergic neurotransmission controlled by cholinergic activity. Similar to SAI, LAI is also reduced by lorazepam but not baclofen, suggesting that it reflects GABA_A_ not GABA_B_ receptor activity (Turco et al., 2018).

Modulation of SAI and/or LAI with exercise may indicate changes in sensorimotor integration or excitability of sensorimotor pathways. Only two studies have used afferent inhibition to assess exercise-induce neuroplasticity. First, Yamazaki et al. (2019) showed that low-intensity pedaling reduced SAI within the exercised TA muscles and non-exercised FDI muscle. Second, Brown et al. (2020) showed that moderate-intensity cycling increased LAI, while SAI was unchanged. Further study replication would be needed to fully elucidate the effects of exercise-induced neuroplasticity on afferent inhibition.

### Cerebellar Inhibition (CBI)

Cerebellar inhibition (CBI) is observed when a TMS pulse delivered to the cerebellum inhibits the MEP evoked by TMS delivered to M1 (Figure 3C). The ISI used to measure CBI is 5–8 ms (Daskalakis et al., 2004; Rothwell, 2011). When delivered to the cerebellum, it has been postulated that TMS activates Purkinje cells, leading to inhibition of M1 via the dentate nucleus (Ugawa et al., 1991; Tremblay et al., 2016). Previously, CBI has been used to investigate the cerebellum's role in motor learning (Jayaram et al., 2011; Schlerf et al., 2012; Baarbé et al., 2014; Spampinato and Celnik, 2018). Importantly, as the CS is delivered to the cerebellum, this will lead to activation of neck muscles (Demirtas-Tatlidede et al., 2011) and potentially discomfort to the participant (Fernandez et al., 2018).

Cerebellar inhibition is typically acquired with TS intensities of 1 mV MEP (Daskalakis et al., 2004; Baarbé et al., 2014) and CS intensities at or just below the maximum tolerated intensity, a value that varies according to TMS coil manufacturers (Spampinato et al., 2020). Alternatively, Baarbé et al. (2014) found that CS intensities evoking 50% inhibition of the MEP (termed CBI_50_) is a sensitive method to detect neuroplastic changes in cerebellar-M1 connectivity with motor learning.

To our knowledge, only one previous study has assessed CBI in the context of exercise. This study evoked CBI using a CS intensity of 100 or 120% RMT and a TS intensity of 1 mV MEP (Mang et al., 2016). A single session of high-intensity cycling reduced CBI in healthy participants (Mang et al., 2016). This suggests that exercise induced a change in the excitability of cerebellar-M1 networks. Disinhibition of the cerebellum (i.e., a reduction in CBI) is observed following motor learning (Jayaram et al., 2011; Schlerf et al., 2012; Baarbé et al., 2014). Therefore, a reduction in CBI following exercise may prime the central nervous system for subsequent motor learning, as suggested elsewhere (Mang et al., 2013, 2016). It is unknown whether long-term training also changes CBI, or if exercise changes CBI in clinical populations.

### Interhemispheric Inhibition (IHI)

Interhemispheric inhibition (IHI) reflects the excitability of transcallosal pathways between bilateral motor cortices. Interhemispheric inhibition can be measured when a suprathreshold CS is delivered to M1 prior to a suprathreshold TS delivered to the opposite M1 (Figure 3D). Short-interval IHI (SIHI) is observed at ISIs of 8–12 ms and long-interval IHI (LIHI) is observed at ISIs of 40–50 ms (Ni et al., 2009). The CS and TS intensities are typically adjusted to a 1 mV MEP (Daskalakis et al., 2002; Kukaswadia et al., 2005). Previous work shows that SIHI reduces I2 and I3 waves (Di Lazzaro et al., 1999a), while the I-wave origin of LIHI has yet to be investigated.

Both baclofen (Irlbacher et al., 2007) and lorazepam (Sommer et al., 2012) increase LIHI, but not midazolam (Irlbacher et al., 2007). Further, SIHI is not modulated by baclofen (Irlbacher et al., 2007; Florian et al., 2008) or the benzodiazepines midazolam (Irlbacher et al., 2007) and diazepam (Florian et al., 2008), but is reduced by carbamazepine (Sommer et al., 2012). This suggests that LIHI reflects GABA_B_ and GABA_A_ receptor activity, while SIHI is reflective of voltage-gated sodium channels. Therefore, SIHI and LIHI may act through distinct neuronal populations.

Interhemispheric inhibition is thought to be important for suppressing unwanted mirror movements (Duque et al., 2005, 2007) and bimanual control (Nelson et al., 2009). Recently, we showed that reduced IHI with muscle contraction is correlated with territorial expansion of that muscle's representation within M1 (Turco et al., 2019). This suggests that IHI may be an important mechanism that modulates M1 organization. To our knowledge, no studies have assessed effects of acute aerobic exercise on IHI, while only one has assessed the effects of chronic aerobic exercise. McGregor et al. (2018) found no change in LIHI following 12 weeks of aerobic cycling in older adults. At present, it is unknown if chronic exercise modulates LIHI in young adults or if chronic exercise modulates SIHI.

## Important Considerations for TMS-Exercise Research

Changes in skin temperature and hydration occur (i.e., perspiration) during aerobic exercise, and these effects can reduce adherence and alter the placement of EMG electrodes (i.e., slippage). Yamazaki et al. (2019) found an increase in skin temperature overlaying the FDI muscle after a session of exercise, but did not assess whether changes in SICI and SAI with exercise were related to changes in skin temperature. Next, perspiration attenuates the surface EMG signal (Abdoli-Eramaki et al., 2012), and can induce shifts in the electrode position that alters the EMG signal (Garcia et al., 2017; Merletti and Muceli, 2019). One solution is to measure the maximal M-wave (M_max_) before and after exercise. The M_max_ represents the recruitment of all motor units innervating a given muscle and is obtained by orthodromic activation of motor efferents following peripheral nerve stimulation given during muscle relaxation. Acute aerobic exercise is not anticipated to change M_max_ since acute exercise-induced effects are likely to be mediated by changes in the central nervous system. For example, most studies have reported no change in M_max_ following an acute bout of exercise (Neva et al., 2017; El-Sayes et al., 2019b; Walsh et al., 2019; Brown et al., 2020; Nicolini et al., 2020). However, there are instances where acute aerobic exercise reduces M_max_ (McDonnell et al., 2013). This reduction is likely to relate to changes in the conductivity that can occur with shifts in electrode position related to perspiration. Therefore, one approach is to obtain measures of M_max_ before and following exercise, and confirm that M_max_ has not change. If M_max_ has changed, another approach is to normalize MEP amplitude to M_max_ as performed elsewhere (McDonnell et al., 2013; Neva et al., 2017; El-Sayes et al., 2019b; Nicolini et al., 2019, 2020; Walsh et al., 2019; Brown et al., 2020).

An important consideration when using TMS paradigms to assess exercise-induced neuroplasticity is the origin of plasticity. While epidural recordings and I-wave modulation provide insight into the spinal vs. cortical origin of TMS measures, it cannot be ruled out that changes in spinal excitability underly the neuroplastic effects induced by exercise. To disentangle spinal vs. cortical locations of neuroplasticity, studies may choose to measure spinal reflexes in addition to TMS measures. For example, H-reflexes and F-waves provide information about spinal motor neuron excitability (McNeil et al., 2013). Overall, using a combination of techniques probing cortical and spinal neural pathways provides greater insight into the mechanisms of exercise-induced neuroplasticity.

Fitness and/or physical activity levels may influence the propensity for exercise-induced neuroplasticity. Lulic et al. (2017) found that acute aerobic exercise only increased MEPs in the high physically active group, but not in the low physically active group as categorized by the International Physical Activity Questionnaire (IPAQ). However, MacDonald et al. (2019) found no correlation between corticospinal excitability change and aerobic fitness as assessed by peak oxygen uptake (VO_2max_). These results suggest that physical activity, but not necessarily cardiorespiratory fitness, is an important consideration for exercise-induced neuroplasticity. Nevertheless, both of these variables would aid in the interpretation of neurophysiological changes following exercise. Therefore, physical activity and/or fitness levels should be well-defined in future studies.

Another important consideration for TMS-exercise research is the timing of post-exercise assessments to determine the duration of neuroplasticity. A number of TMS exercise studies have implemented multiple post-exercise assessments of TMS measures (Singh et al., 2014; Smith et al., 2014; Mooney et al., 2016; Neva et al., 2017; MacDonald et al., 2019; Walsh et al., 2019; Yamazaki et al., 2019; Brown et al., 2020). For example, Mooney et al. (2016) found that LICI decreased 10 and 20 min after acute exercise but returned to baseline levels 30 min after exercise. This demonstrates that an acute bout of aerobic exercise does not induce neuroplasticity for an extended period of time. Further, multiple post-intervention assessments may capture delayed changes in neurophysiology. For example Smith et al. (2014) only found a decrease in SICI post 15 min and not immediately post the exercise. Therefore, future studies should carefully consider the number and timing of post-exercise TMS assessments to capture delayed neuroplastic effects and the duration of neuroplastic effects.

Future research should consider investigating exercise-induced neuroplasticity as a function of participant demographics. One study has showed that both males and females demonstrate increased MEP recruitment curves and reduced SICI after one session of aerobic exercise, regardless of menstrual cycle phase (El-Sayes et al., 2019b). However, the influence of biological sex and ovarian hormones on chronic aerobic exercise effects are unknown. Further, it is unknown if neuroplasticity can be induced by exercise within different age groups. With the exception of McGregor et al. (2018), all studies within Table 1 recruited participants with mean ages of 20–35 years. McGregor et al. (2018) showed that chronic aerobic exercise can increase iSP in older adults, but does not change LIHI. Further, McGregor et al. (2013) showed that the iSP is longer is physically fit compared to sedentary middle-aged adults. Harasym et al. (2020) reported no difference in single- (RMT, AMT, MEP recruitment curve) and paired-pulse (SICI, ICF, SAI, LAI, SIHI, LIHI) TMS measures between sedentary and active postmenopausal women. However, it is unknown if an acute bout of aerobic exercise is capable of inducing neuroplasticity within older participants. In addition, studies should consider acquiring paired-pulse TMS measures with different coil directions. First, compared to the more commonly used posterior-anterior current (PA), the anterior-posterior (AP) current activates different neuronal populations (Ni et al., 2011), providing insight into the I-wave generating interneurons that may be modulated following exercise. Second, the AP current evokes stronger SICI in older compared to younger adults, while the PA current evokes similar SICI between the two (Sale et al., 2016). Therefore, when investigating exercise-induced neuroplasticity across age groups, acquiring data in various current directions may lead to differing results.

Finally, future research may want to consider implementing reliability analyses within experimental designs. TMS measures of corticospinal excitability are subject to variability from multiple sources including, but not limited to, circadian factors (Lang et al., 2011; Bocquillon et al., 2017), variations in coil positioning (Koski et al., 2005; de Goede et al., 2018), and participant attention and fatigue (Stefan et al., 2004; Kotb et al., 2005; Kreuzer et al., 2011). The majority of studies reviewed herein employed a no-exercise control condition to confirm that were no significant changes in TMS measures due to the passage of time (McDonnell et al., 2013; Mang et al., 2014, 2016; Mooney et al., 2016; Stavrinos and Coxon, 2017; Smith et al., 2018; Opie and Semmler, 2019; Yamazaki et al., 2019; Andrews et al., 2020; Brown et al., 2020; Morris et al., 2020; Nicolini et al., 2020). However, future studies may wish to use these no-exercise conditions as an opportunity to determine the reliability of TMS assessments and increase the validity of their findings. For example, relative reliability assessments such as the intraclass correlation coefficient (ICC) would be useful in determining the test-retest reliability of a measurement (Koo and Li, 2016) and absolute reliability metrics such as the Standard Error of Measurement (SEM) can be computed to quantify measurement error (Weir, 2005). Further, the Smallest Detectable Change (SDC), defined as minimum change in a measurement that is beyond measurement error and is considered to be a “real” change (Weir, 2005; Beaulieu et al., 2017), can be calculated at the individual (SDC_individual_) and group level (SDC_group_) (Schambra et al., 2015). Specifically, the SDC_group_ of a measure can be calculated from the no-exercise condition and be used as a complement to hypothesis testing (Schambra et al., 2015). As an example, a study may assess the change in RMT following 20 min of aerobic cycling (exercise condition) or 20 min of rest (control condition). Calculations from the data within the control condition reveal that SDC_individual_ is 10% MSO and SDC_group_ is 3% MSO. This indicates that an *individual* would need to exhibit a change in RMT >10% MSO in the exercise condition to confidently conclude that said individual showed real physiological change. In contrast, the *group-averaged mean* would need to exhibit a change in RMT greater than only 3% MSO in the exercise condition to confidently conclude a “real” change exceeding measurement error. Overall, reporting these metrics of reliability alongside means comparisons can increase confidence that exercise is or is not inducing real neuroplastic change in the nervous system.

## Conclusion

This review aimed to summarize the physiology and methodology of single- and paired-pulse TMS paradigms that can be used to study exercise-induced neuroplasticity, and to highlight the previous research that has studied how these measures change following aerobic exercise. Future studies using TMS to investigate exercise-induced neuroplasticity should take into consideration important variables that could influence data interpretation including changes in electrode conductance and muscle contractility with exercise, spinal contributions to neuroplasticity, physical fitness, age, reliability, and the timing of TMS assessments.

## Author Contributions

CVT conceived, wrote, edited, and finalized manuscript. AJN conceived, edited, and finalized manuscript.

## Conflict of Interest

The authors declare that the research was conducted in the absence of any commercial or financial relationships that could be construed as a potential conflict of interest.

## Abbreviations

*AMT*: active motor threshold
*AP*: anterior-posterior
*APB*: abductor pollicis brevis
*CBI*: cerebellar inhibition
CBI_50_: conditioning stimulus intensity resulting in 50% inhibition of the MEP
*CS*: conditioning stimulus
*CSP*: contralateral silent period
*DA*: dopamine
*D-wave*: direct wave
*ECR*: extensor carpi radialis
*EMG*: electromyography
*FDI*: first dorsal interosseous
*GABA*: gamma-aminobutyric acid
*HICE*: high-intensity continuous exercise
*HIIE*: high-intensity interval exercise
*HIIT*: high-intensity interval training
*HR*_*max*_: maximal heart rate
*HRR*: heart rate reserve
*ICF*: intracortical facilitation
*IHI*: interhemispheric inhibition
*IPAQ*: International Physical Activity Questionnaire
*ISI*: interstimulus interval
*iSP*: ipsilateral silent period
*I-wave*: indirect wave
*LAI*: long-latency afferent inhibition
*LICE*: low-intensity continuous exercise
*LICI*: long-interval intracortical inhibition
*LIHI*: long-interval interhemispheric inhibition
*M1*: primary motor cortex
*MEP*: motor-evoked potential
*MEP*_*max*_: maximum MEP amplitude
*MIAT*: moderate-intensity aerobic training
*MICE*: moderate-intensity continuous exercise
*MIIE*: moderate-intensity interval exercise
*ML-PEST*: maximum likelihood parameter estimation by sequential testing
*M*_*max*_: maximal M-wave
*MRS*: magnetic resonance spectroscopy
*MSO*: maximum stimulator output
*MT*: motor threshold
*MTI*: maximum-tolerated intensity
*MVC*: maximum voluntary contraction
*NA*: noradrenaline
*NMDA*: N-methyl-D-aspartate
*PA*: posterior-anterior
*PD*: Parkinson's disease
*RMT*: resting motor threshold
*RPE*: rating of perceived exertion
*SAI*: short-latency afferent inhibition
*SCI*: spinal cord injury
*SICF*: short-interval intracortical facilitation
*SICI*: short-interval intracortical inhibition
*SIHI*: short-interval interhemispheric inhibition
*SNAP*: sensory nerve action potential
*ST*: sensory threshold
*TA*: tibialis anterior
*TES*: transcranial electric stimulation
*TMS*: transcranial magnetic stimulation
*TS*: test stimulus
*VO*_2*max*_: peak oxygen uptake
*W*_*peak*_: peak power output.
